# Autistic Self-Advocacy and the Neurodiversity Movement: Implications for Autism Early Intervention Research and Practice

**DOI:** 10.3389/fpsyg.2021.635690

**Published:** 2021-04-12

**Authors:** Kathy Leadbitter, Karen Leneh Buckle, Ceri Ellis, Martijn Dekker

**Affiliations:** ^1^Division of Neuroscience and Experimental Psychology, University of Manchester, Manchester, United Kingdom; ^2^The Autscape Organisation, Coventry, United Kingdom; ^3^The European Council of Autistic People, Prague, Czechia

**Keywords:** autism, children, neurodiversity, self-advocacy, early intervention

## Abstract

The growth of autistic self-advocacy and the neurodiversity movement has brought about new ethical, theoretical and ideological debates within autism theory, research and practice. These debates have had genuine impact within some areas of autism research but their influence is less evident within early intervention research. In this paper, we argue that all autism intervention stakeholders need to understand and actively engage with the views of autistic people and with neurodiversity as a concept and movement. In so doing, intervention researchers and practitioners are required to move away from a normative agenda and pay diligence to environmental goodness-of-fit, autistic developmental trajectories, internal drivers and experiences, and autistic prioritized intervention targets. Autism intervention researchers must respond to these debates by reframing effectiveness, developing tools to measure autistic prioritized outcomes, and forming partnerships with autistic people. There is a pressing need for increased reflection and articulation around how intervention practices align with a neurodiversity framework and greater emphasis within intervention programmes on natural developmental processes, coping strategies, autonomy, and well-being.

## Introduction

The last two decades have brought about huge socio-political shifts within the world of autism theory, research and practice. In the mid-1990s, the emergence of the internet provided a more accessible text-based means of communication and empowered a growing number of autistic people to connect and share ideas with one another (Dekker, 2020)^1^. Out of the early autistic social groups of the 1990s emerged autistic culture, the autistic self-advocacy movement, and the assertion that autism is a valid way of being. This environment also gave rise to the neurodiversity movement (Singer, 1998). Through the 2000s, the neurodiversity movement has been galvanized in a large part due to the voices, advocacy and protest of the autistic community, facilitated through developments in online communication and networks (Kras, 2009) and is increasingly influencing academic, clinical and lay understanding of autism and other forms of neurological difference.

A central premise of the neurodiversity movement is that variations in neurological development and functioning across humans are a natural and valuable part of human variation and therefore not necessarily pathological (e.g., Jaarsma and Welin, 2012; Kapp, 2020). Neurodiversity as a social justice and civil rights movement intersects with the wider disability rights movement (Hughes, 2016). The most significant premise of both is that disability is not simply a defect in the individual, but arises from the interaction between a non-standard individual and an unaccommodating environment (the social model of disability; Oliver, 1990). Consistent with this stance, many neurodiversity proponents do view autism as a disability. From this theoretical underpinning, the neurodiversity movement makes several demands, including the recognition and acceptance of the value of cognitive variation as a form of biodiversity and hence its positive contribution to groups, communities and societies (the social-ecological perspective; Chapman, 2020) and equal rights leading to an end to discriminatory policies and practices (Runswick-Cole, 2014).

The amplification of autistic viewpoints, coupled with the traction of neurodiversity as a concept and movement, has led to the emergence of new ethical, theoretical and ideological debates. These debates and discussions have had genuine impact within some areas of autism research, predominantly that focused on adults. Examples of this impact include: (a) debates over whether the social difficulties experienced by autistic people are best understood as being a problem within the individual, or a problem between two (mis-matched) individuals, and the resulting research into the Double Empathy Problem and diversity in social intelligence (Milton, 2012; Crompton et al., 2020); (b) calls from the autistic community for a greater emphasis on improving mental health and quality of life in autistic individuals (Autistica, 2015; National Autistic Taskforce, 2019) and an increase in research into effective, person-centered mental health interventions (e.g., Crane et al., 2019; Cassidy et al., 2020; Parr et al., 2020) and (c) research into community preferences over the language used to describe autism and autistic people (e.g., Kenny et al., 2016; Bury et al., 2020). Despite these impacts within adult-focussed research, these debates are still rarely directly addressed in early intervention research, where the impact of the autistic viewpoint is often implicit or not present at all. The absence of clear and proactive engagement with these debates contributes to a lack of confidence in an evidence base that has already shaky foundations due to its poor methodological quality and widespread unreported conflicts of interest (e.g., French and Kennedy, 2018; Bottema-Beutel et al., 2020; Sandbank et al., 2020). In this paper, we argue that all autism intervention stakeholders need to understand and actively engage with these debates. We focus on psychosocial intervention programmes that aim to improve aspects of young autistic children's cognitive, behavioral, emotional, or relational functioning and reflect on the purpose of early autism intervention, the types of intervention methods we use, and how these align with the priorities of autistic people. We then reflect upon issues pertinent to research into early autism intervention and the challenges and opportunities presented by these shifts, pointing to future directions.

## Implications for Early Autism Intervention

Whilst there is agreement amongst most of the autistic community, clinicians, and researchers that interventions should be available to help autistic people of all ages to thrive and reach their potential (UK Parliament, 2020), there are many controversies surrounding what this means in practice. Until the 1990s, it was common to consider therapy for autistic children as a means of reaching the child within their “autistic shell” and drawing them out, resulting in a normal or near-normal child (e.g., Park, 1972; Kaufman, 1976; Maurice, 1998). The earliest actions of the autistic self-advocacy movement were to call for the recognition of autism as an essential aspect of the person (Sinclair, 1993). Autistic self-advocates opposed early autism interventions with a stated treatment goal to make a child no longer, or less, autistic. However, some stakeholders, in particular parents of autistic children with substantial intellectual, language and behavioral challenges, argued that autistic adults without these challenges could not speak to their children's experience, and that their children required such interventions in order to achieve a reasonable quality of life (Dekker, 2017; Fletcher-Watson, 2018). This disagreement has yet to be fully resolved. Some activists continue to argue that any attempt to alter an autistic person is misguided, thereby rejecting any form of early intervention (e.g., Stevenson, 2015). Some autistic people, parents or other stakeholders continue to oppose neurodiversity as a concept or social movement, arguing, for example, that it presents a sanitized view of autism, excludes those with significant language or intellectual disability, and deflects resources from those most in need of support (Happé and Frith, 2020; Hughes, 2020).

Objections to neurodiversity are often based on an erroneous conception of the tenets of the movement (den Houting, 2019). Fundamentally, neurodiversity emphasizes the collective strength inherent in cognitive diversity (Chapman, 2020) and that this strength arises from all kinds of differences, including those associated with autism, intellectual disability or language impairment (Kapp, 2020). Moreover, neurodiversity activism, which includes some non-speaking activists, specifically includes and advocates for those who are unable to do so themselves. A balanced view of neurodiversity recognizes that, whilst diversity brings fundamental collective advantages, within any one neurodivergent individual weaknesses are often the inextricable partner of strengths, and that individuals can want things to be different and still want to be themselves. It includes the understanding that some neurological differences are disadvantageous, either inherently or in interaction with the environment, and could benefit from correspondingly targeted intervention.

Adopting this balanced account of neurodiversity, we can derive three important implications for intervention. Firstly, neurodiversity-informed intervention opposes any attempt to “cure” or “normalize” autistic children, and, whilst in many contexts this talk is no longer acceptable (Happé and Frith, 2020), there are still many interventions purporting an explicit or implicit curative or normative agenda (Mottron, 2017). This opposition is conceptual: even if it were desirable, it would not be possible to cure someone of an innate neurological difference. It is also existential: autism is so pervasive and profound, that attempts to target autism itself fundamentally changes the person; many autistic people have equated being cured of autism as tantamount to death, as they would be a completely new individual (Sinclair, 1993). There is also increasing evidence to support opposition on ethical grounds as: (a) this approach leads to individuals “masking” their autism or attempting to “pass” as neurotypical at a huge cost to their mental health and well-being (Milton and Moon, 2012; Mandy, 2019) and (b) many intervention programs attempt to teach “normative behavior” without referencing empirical evidence for what “normative behavior” looks like and thereby teach autistic children to behave in ways that do not actually resemble autistic or non-autistic children (Bottema-Beutel et al., 2018).

Secondly, interventions informed by neurodiversity do carefully address any extrinsic factors around an autistic child that contribute to disadvantage and negative experiences and therefore aim to improve the “goodness of fit” between the child and their physical or socio-emotional environment (Lai and Szatmari, 2019). Interventions that encourage and provide opportunities for physical, sensory and emotional regulation (e.g., sensory integration therapy, Randell et al., 2019) are compatible with this stance. Interventions can promote an understanding of autism and neurodiversity in people in the child's world, such as caregivers (e.g., EMPOWER-ASD intervention,^2^ Systemic Autism-related Family Enabling intervention, McKenzie et al., 2019; SOLACE programme, Lodder et al., 2020), and education professionals and peers (e.g., Learning about Neurodiversity at School project^3^). These interventions also support non-autistic people to build resilience, develop a positive philosophy toward the autistic child, and to build relationships in a respectful, supportive and harmonious manner. Targeted interventions for autistic children can also build effective communication between the child and others, for example, by coaching caregivers and education professionals to “speak the child's language” (e.g., Paediatric Autism Communication Therapy; Pickles et al., 2016; Green et al., 2018). Other interventions aim to support neurodivergent children by working with them directly to understand their autism and build self-awareness and self-esteem (e.g., Pegasus, Gordon et al., 2015; the Spectacular Girls programme^4^). Intervention efforts that target the child's environment may address early external causes of distress (e.g., non-acceptance/non-accommodation of needs, bullying, and exclusion) and therefore help to prevent future mental health problems.

A third implication for interventions concerns those aspects of autism that are disadvantageous in and of themselves. A balanced view of neurodiversity mandates that specific characteristics of autism be depathologised, unless those characteristics cause harm or discomfort to the individual or a violation of others' rights. The complexity for autism interventions concerns the fine line between supporting a child's development and attempting to change the essence of the person. It also concerns the fine balance between accommodation of autistic behaviors and the alleviation of actually or potentially detrimental cognitive or behavioral phenomena. This balance is challenged by differing opinions as to what constitutes and causes suffering, and difficulties in ascertaining the views of individual children due to their young age, communication difficulties, and lack of understanding of potential future consequences. There are no simple solutions to these complexities. However, there are some principles that can guide us in a direction that is consistent with autistic viewpoints and a neurodiversity stance.

### Consideration of Internal Drives and Experiences

A key principle concerns looking beyond observable behavior to consider internal drives and experiences. An under-appreciation of the sensory and emotional experiences of neurodivergent children can result in attempts to reduce or eliminate natural coping and self-regulation strategies, such as repetitive motor mannerisms or “stimming” behaviors (Bascom, 2012; Kapp et al., 2019). Eliminating such behaviors can lead to children being unable to avoid aversive experiences, calm themselves, or to communicate intense emotions (Kapp et al., 2019). Moreover, there is increasing evidence that different developmental routes can lead to the same outcome, whereby atypical developmental processes are actually beneficial to that individual's intrinsic developmental trajectory; examples are echolalia and hyperlexia as alternative routes into functional spoken language (Mottron, 2017). Focussing on reduction of the behaviors that define the autism diagnosis fails to consider that these behaviors are the outcome of different underlying neurology and interfering with them may undermine a child's natural coping strategies and development. Early interventions should therefore work with (not against) the child's developmental trajectory, as well as with their natural way of learning (Fletcher-Watson, 2018).

### Re-evaluation of Intervention Targets

We should evaluate the motivations driving the decisions around intervention targets and not assume that the things that make a good neurotypical life are identical to autistic priorities (Buckle, 2013; Milton, 2014; Iemmi et al., 2017). Active listening to the autistic community helps understand autistic priorities around intervention targets, as does close attention to research that highlights the phenomena that cause autistic people difficulty or distress, affect quality of life and for which autistic people actively ask for support. Examples include autistic inertia (Buckle et al., 2020), life skills (Pellicano et al., 2014), intolerance of uncertainty (Rodgers et al., 2018), and anxiety (Robertson et al., 2018). Certainly, avoiding intervention techniques that themselves cause emotional harm is crucial, and a key underlying principle is to support the autistic child's ability to exert choice and control in their life as they develop.

### Emphasis on Strengths, Pleasure, and Well-Being

Interventions should respect and enhance those things that bring happiness and joy. Passionate interests can bring pleasure and relaxation through repetition or intensity of immersion in tasks, behaviors or objects (e.g., autistic reflections on flow states; Murray et al., 2005; McDonnell and Milton, 2014). Predictable access to preferred activities not only decreases expressions of negative emotions (sometimes manifest as “challenging behavior”) but also can provide opportunities for expertise and genuine social bonding (Mottron, 2017; Grove et al., 2018; Wood, 2019). The adoption of a positive psychology and strengths-based stance (Burnham Riosa et al., 2017; Dykshoorn and Cormier, 2019) refocuses intervention efforts away from reducing deficits and toward enhancing those activities or skills that naturally lead to learning, social connection, and well-being. Intervention efforts should leave alone unconventional characteristics that cause no harm to self or other, such as a monotone voice or preference for solitude. Lifespan research into the childhood factors that are associated with long-term well-being will enable us to boost these important factors through early intervention (Rodogno et al., 2016; Pickles et al., 2020).

### Promotion of Autonomy

The final fundamental principle concerns autonomy and the right to say “no”. Poignant accounts from autistic adults describe the use within early interventions of overbearing physical prompting, ignoring of communication attempts, or outright removal of their right to communicate “no” and how this left them passive, traumatized, and vulnerable to abuse (Kirkham, 2017; McGill and Robinson, 2020). These practices must be avoided. Autonomy is essential to creating the life one wants to lead (National Autistic Taskforce, 2019; Späth and Jongsma, 2020). In order to achieve any significant level of autonomy, one must have functional communication, so interventions supporting communication (not simply speech) and understanding required for the expression of autonomy are justified, as long as they are undertaken ethically, with true respect for the individual.

## Application of the Neurodiversity Framework to Autism Intervention Research

### Re-Framing Effectiveness

Early intervention researchers understand the importance of an evidence base and effectiveness is often the key factor when evaluating evidence. Clearly, it is critical that research informs us about intervention effectiveness – no one wants to spend limited resources on interventions that do not work. However, effectiveness needs to be understood within the context of the above principles. While an intervention may be effective at reducing autistic behavior, if it leaves the child without coping mechanisms or at risk of mental health difficulties, it has not been effective in improving their life. We need to reframe effectiveness to concentrate on the outcomes that are most important to the long-term well-being and autonomy of the children involved and the preferences and priorities of autistic people (Neumeier and Brown, 2020); research can then evaluate the extent to which these prioritized outcomes are (or are not) improved by any particular intervention.

### Outcome Measurement

The landscape of tools used to measure intervention outcomes is strongly focused on the reduction of autism symptoms (e.g., Provenzani et al., 2020). Conceptually, this falls squarely within a normalization agenda: if children's autistic behaviors are reduced sufficiently, they will no longer meet the criteria for autism. In practice, autism symptomatology as a metric amalgamates many different variables. Many of these target variables are incompatible with a balanced view of neurodiversity, such as imposing non-autistic social behaviors or reducing sensory behaviors or motor mannerisms that act as coping strategies. However, others are consistent with it, e.g., improving communication (Kapp, 2020). As a discipline, we need to move measurement away from autism symptomatology and produce validated tools that assess the goodness of fit between an individual and their social, emotional and physical environment. There are good examples already, such as the Autism Five Minute Speech Sample that measures the emotional climate around the autistic child (Benson et al., 2011), and the Dyadic Communication Measure for Autism that assesses caregiver communicative synchrony (Aldred et al., 2004; Green et al., 2010). However, additional measures of environmental outcomes are needed. We also need robust and creative ways to measure, in children with all levels of communication ability, specific and transparent intervention outcomes that are verifiably beneficial, including autonomy, quality of life and the variables that easily impact on these, such as functional communication, inertia, and anxiety (McConachie et al., 2015). The International Classification of Functioning, Disability and Health's “Core Set for Autism Spectrum Disorder” (Bölte et al., 2014) assesses such outcomes within clinical contexts and could be developed for use within intervention research.

### Partnerships With Autistic People

In the UK there is now an increased understanding amongst researchers and funding bodies of community priorities (e.g., James Lind Alliance, 2016) and more meaningful involvement of autistic people in research (Pellicano et al., 2014; Fletcher-Watson et al., 2019). More neurodivergent/autistic people are leading academic discourse (e.g., Chapman, 2020; Kapp, 2020) and empirical studies (e.g., Belcher et al., 2019; Buckle et al., 2020) and there is a greater emphasis on participatory and action research models with autistic viewpoints and experiences at the center (e.g., Crane et al., 2019; Lam et al., 2020). These developments have cast light on the need for autism researchers to re-align their priorities and rethink some of the ways in which they work, thereby slowly changing the emphasis and tone of research.

Parents have historically been the default channel for meaningful involvement within research, and trials of early interventions still typically center on parental views and priorities (e.g., Leadbitter et al., 2018). Although they have their child's best interests at heart, neurotypical parents may be missing essential aspects of understanding from their autistic child's perspective. One argument put forward against involving autistic adults in child-focused research is that articulate and intelligent autistic adults cannot speak for the experience of children with significant intellectual or language disability. We need to be much more invested and creative in exploring ways to garner and document the views of children and adults who have severe communication impairments and this is an important focus for future research (Happé and Frith, 2020). We also need to recognize that autistic adults often bring valuable expertise to child-focused research. Autistic people can speak to what a good autistic life is like (Iemmi et al., 2017) and what might have helped them. Many autistic self-advocates are parents of non-speaking children or were such children themselves. Some autistic people are well-connected with others and can draw on a wide range of experiences. Researchers can also become better acquainted generally with autistic viewpoints through the sentiments actively, and often passionately, shared by autistic people in general forums. It is easier than ever before for neurotypical researchers to access and understand autistic culture and preferences through books, blogs, video accounts, and social media posts.

## Conclusions and Future Directions

Autistic self-advocacy and the neurodiversity movement offer up valuable opportunities to autism intervention practitioners and researchers. A balanced neurodiversity stance offers key principles to steer the development, delivery and evaluation of early interventions. Future directions for research and practice include: (1) partnerships with autistic people, alongside caregivers and other stakeholders, on intervention research steering and advisory boards and throughout engagement, involvement and co-production processes; (2) reflection by intervention researchers and practitioners upon how their intervention practices align with a neurodiversity framework and the views of autistic people, particularly around intervention targets and methods, and more transparent articulation of these issues in engagement and dissemination activities; (3) greater regard within intervention programmes to natural autistic developmental processes, coping strategies, autonomy and well-being; and (4) increased efforts to develop and validate tools to measure autistic prioritized outcomes and the goodness-of-fit between an autistic individual and their environment. With close attention to the needs, preferences and priorities of autistic people, we can move beyond historical divides, misunderstandings and wrongdoings to a place where we value the expertise of autistic people, embrace practices that respect and accept individual neurotypes, and ensure our interventions address the things that matter most to the recipients.

## Data Availability Statement

The original contributions presented in the study are included in the article/supplementary material, further inquiries can be directed to the corresponding author.

## Author Contributions

All authors contributed to the development of ideas and viewpoint, the review of the literature, and writing the article.

## Conflict of Interest

The authors declare that the research was conducted in the absence of any commercial or financial relationships that could be construed as a potential conflict of interest.

## Footnotes

^1^We use identity-first language in keeping with the preference of many autistic people (Kenny et al., 2016). We also use the more neutral term “autism” rather than “Autism Spectrum Disorder”.

^2^www.reach-asd.org.

^3^https://dart.ed.ac.uk/research/leans/.

^4^https://helenclarkeautism.com/spectacular-girls.
